# How Social Situations Affect the Relationships Between Academic Emotional Suppression and Expression and Likability Among Adolescents

**DOI:** 10.3390/bs14121184

**Published:** 2024-12-11

**Authors:** Ying Liu, Biao Sang, Xiaoyun Chai

**Affiliations:** 1 Wenbo College, East China University of Political Science and Law, Shanghai 200042, China; yingliu@ecupl.edu.cn; 2 Lab for Educational Big Data and Policymaking, Shanghai Academy of Educational Sciences, Shanghai 200032, China; 3 School of Psychology and Cognitive Science, East China Normal University, Shanghai 200062, China; 4 Department of Applied Psychology, Hubei University of Medicine, Shiyan 442000, China; xy_chai@hbmu.edu.cn

**Keywords:** adolescents, social situation, academic emotion, emotional suppression, emotional expression

## Abstract

Social situation is an important factor in determining whether or not individuals express emotions and how effectively they express them, but few researchers have explored its role (e.g., with others of varying degrees of intimacy and hierarchy) in the social outcomes of academic emotional suppression and expression. Relationships between the adolescents’ suppression and expression of emotions and their likability in social situations involving a range of people (e.g., classmates, good friends, teachers) were examined in the current study. A total of 120 adolescents and 74 teachers were selected for this investigation, the results indicating a difference in an individual’s likability when suppressing and expressing academic emotions in the presence of others. Specifically, expressing academic emotions in the presence of good friends achieves a higher level of likability than in the presence of classmates; furthermore, suppressing negative academic emotions in the presence of classmates garners a higher level of likability. Adolescents who express their positive and negative academic emotions in the presence of good friends can get higher likability. Teachers prefer adolescents who express positive academic emotions in their presence. These findings emphasize the importance of social situations in the use of strategies to regulate academic emotion regulation and verify the adaptability of emotional regulation.

## 1. Introduction

Emotional regulation, which is closely linked to individual mental health [1,2,3], is the process by which individuals influence what emotions they have, when they have them, and how they experience or express them [4]. In this process, individuals can change their emotional experience by adopting a variety of strategies, among which emotional suppression and expression are commonly used in the social process [5,6]. Through emotional suppression, individuals can consciously hide their emotional experience, and through emotional expression they can consciously reveal their emotional experience [7,8]. Previous researchers have discovered that both suppression and expression of emotions have important implications for the physical and mental development of individuals [9,10,11], but they have, to some degree, overlooked the role that situation (the environmental conditions that influence the occurrence of events or individual behavior) [12] plays in them. According to more and more theoretical and empirical studies, the effectiveness of strategies to regulate emotion varies from situation to situation; furthermore, successful regulation of emotion involves individuals flexibly choosing and using those strategies according to the situation [7,13,14,15]. Few researchers, however, have explored the role of situation in the social consequences of emotion regulation, especially the regulation of academic emotion. The aim of this study was, therefore, to examine the social outcomes of adolescents’ academic emotional suppression and expression in various social contexts.

### 1.1. The Importance of Situation in Social Outcomes of Emotional Suppression and Expression

Previous researchers on emotional suppression have focused more on its impact on individuals and less on its social consequences [16]. Few studies concerned with social consequence have also suggested that emotional suppression may have adverse effects on interpersonal relationships, such as poorer first impressions and peer relationships [17,18]. In comparison, emotional expression exhibits more adaptive functions in social interaction (e.g., more popular, more sociable) [19,20,21]. Emotional suppression and expression are not generally beneficial or harmful, but their effectiveness tends to depend on other factors like the situation [7,22].

Flexibility in emotional regulation is the ability of individuals to be flexible in deploying regulatory strategies according to changing situational demands. If the emotional regulation system cannot flexibly respond to immediate situational demands, nonadaptive consequences (e.g., less positive emotions, less popular) may follow [7,23]. More specifically, the strategy–situation fit theory under the framework of the flexibility of emotional regulation holds that the adaptive matching between emotional regulation strategies and situations can maximize the effectiveness of regulation and bring beneficial effects to individuals, while the mismatch of strategies and situations may produce adverse results [7,13,24]. Based on these theories, the situation may indeed influence the regulating effect of the suppression and expression of emotion [7,25,26]. Thus, in some situations, like the existence of a social hierarchy [27] or a mismatch of emotions and situations [28], people may prefer suppression over expression because suppression can be a socially useful strategy for emotional regulation [19,29].

### 1.2. The Importance of Social Situations in the Social Outcomes of Academic Emotional Suppression and Expression

Individuals encounter a variety of social situations in their daily lives, such as inevitable interaction with others. During social interaction, the behavior and performance of the individual are influenced by others, the degree of influence often depending on the number, importance, and proximity of others involved [30]. For example, researchers have found that the presence of peers and parents is an important factor in children’s risk inclinations [31,32], and the presence of strangers can influence the creative performance of college students [33]. Social situations (e.g., presence of other people) can have a significant impact on the behavior of individuals.

As part of daily life, individuals face numerous people in the process of learning, including classmates, friends, family, and teachers. Understanding the impact of such social situations on individuals in the academic field is important. One of the effects is in the area of emotional regulation. Previous studies have suggested that emotional regulation usually occurs in social situations [34], so the social characteristics of the situation (e.g., with whom) could be an important factor in determining how individuals perform that regulation [35].

In social situations, the suppression and expression of emotions are two more commonly used regulation strategies [19]. From the perspective of the social function of emotion, whether and how emotions are expressed affect the individual’s social relationships and social status [20,36]. Because people view emotions as social information, they infer feelings, personality traits, internal psychological logic, and intentions of individuals based on their emotional states [20,37,38,39]. At the same time, individuals also decide whether to express emotions based on their emotional regulation goals (e.g., social goals) [40]; whether expressing certain emotions is beneficial to their social goals depends in part on their relationship with the person with whom they interact [19].

An important index for determining the type of social situation an individual is in is the intimacy of the individual’s relationship with others [41]. Previous studies have suggested that individuals in both Eastern and Western cultures are accustomed to regulating emotional expression based on relationship intimacy [29,42]. For example, Western culture emphasizes that individuals decide whether or not to express emotions based on their level of closeness to others [43]. In Eastern cultures, Confucian-influenced Chinese often conducts social interactions according to the “principle of favouring the intimate” [44], which means that people can express their emotions more honestly and freely to their family and good friends than to others. In addition, East Asian culture emphasizes the distinction between peers and superiors [45]. In Confucianism, another principle of Chinese social interaction is the “principle of respecting the superior” [44], which emphasizes respect for superiors, teachers, and other people of higher status. These two criteria enable individuals to learn to regulate their behavior and emotional expression prudently in front of people at various levels of intimacy or hierarchy [46].

Previous researchers have found that expressing emotions to others with varying levels of intimacy and hierarchy may produce diverse social outcomes. For example, expressing emotions in the presence of ordinary people can have negative consequences. Sommers [47] discovered that college students who expressed negative emotions were less popular and engaged in fewer social activities than those who did not express negative emotions. Compared to friend and couple relationships, people are less willing to express their emotions in exchange (business) relationships and less inclined to like peers who do so [43], but expressing emotions in front of others in intimate relationships may have adaptive consequences. In fact, Pollastri et al. [21] found that high school boys who expressed more emotions to their friends experienced increased social connection with them and in turn experienced more social support and less loneliness; expressing more emotions to others than close ones, however, did not produce similar results. For the social consequences of expressing emotions to others at various levels of hierarchy, one longitudinal study indicated that employees who expressed more positive emotions received more supervisory support 18 months later [48].

In addition, the social outcomes of suppressing emotions in the presence of others with diverse levels of intimacy and hierarchy vary. In the presence of people higher in the hierarchy or outside intimate relationships, suppressing emotional expression is considered to be a situational adaptive behavior [42]. In the presence of family and good friends, suppression of emotions (e.g., happiness) may lead to family dysfunction and poor quality of intimate relationships [49]. For example, the more people suppress their emotions in romantic relationships, the less genuine they feel and, in turn, the worse their happiness and the quality of romantic relationships [50]. Cameron and Overall [16] found that adults who used more emotional suppression in the presence of their partners experienced lower levels of acceptance, closeness, and relationship satisfaction; but emotional expression increased feelings of acceptance and closeness. A study of Chinese college students showed that suppression of happiness was less positively correlated with depression and loneliness and less negatively correlated with social acceptance in the presence of teachers than in the presence of close others (family members, good friends) [42]. Evidently, in line with the strategy–situation fit theory under the framework of the flexibility emotional regulation, practical studies have also demonstrated that in the face of interaction objects with ever-changing intimate relationships and hierarchy levels, individuals need to be flexible in their choice and use these two strategies to achieve close interpersonal relationships [29,51,52]. Although previous studies have provided support for differences in the social adaptability of emotional suppression and expression in the presence of a range of people in a range of social situations, few researchers have explored the characteristics of such differences within academic contexts in depth; furthermore, research in this area remains of important theoretical and practical value.

### 1.3. The Present Study

The purpose of the current study was to explore the social outcomes of suppression and expression of academic emotions in social situations with the presence of a variety of others (classmates, good friends, teachers) with adolescents as participants and academic settings as the background. The reason for choosing adolescents as participants is that their need for emotional regulation increases as the source of stress, emotional sensitivity, and emotional responsiveness increases [53]. Focusing on classmates and good friends is the result of the gradual expansion of the social networks of adolescents, their increasing dependency on peer groups [54,55], and the growing impact of peer relationships on them [56]. Adolescents must develop and use a variety of regulatory strategies to promote positive interaction with one another in order to gain a high degree of acknowledgment from peers [57]. Although rigid use or excessive reliance on any regulation strategy may be nonadaptive [58], using emotional suppression and expression strategies flexibly in the presence of peers is more important [57]. Focusing on teachers is the result of the emphasis of traditional Chinese culture on the concept of “respecting teachers”. To some extent, teachers represent authority in the eyes of students [59], and a good teacher–student relationship is a protective factor in the development of adolescents [60]. Thus, they must establish a good social and emotional connection with teachers [61] to promote their own development [60,62]; the appropriate use of strategies for the regulation of emotion in the presence of teachers is, in fact, one of the ways to promote a good teacher–student relationship [63]. In addition, likability, defined as the degree to which an individual is liked by others [64], is an important indicator of whether an individual has good interpersonal relationships [57]. We, therefore, focused this study on the relationship between emotional suppression and expression of adolescents and their likability in social situations involving a variety of people (classmates, good friends, teachers).

Although no previous researchers have directly addressed this question, based on other relevant studies, we proposed the following hypotheses: (a) Expressing positive and negative academic emotions in the presence of classmates will result in a lower level of likability, but suppressing positive and negative academic emotions will result in a higher level of likability; (b) expressing positive and negative academic emotions in the presence of good friends will result in a higher level of likability, but suppressing positive and negative academic emotions will result in a lower level of likability; and (c) compared with expressing positive academic emotions, suppressing them in the presence of teachers will result in a lower level of likability; compared with expressing negative academic emotions, suppressing these emotions in the presence of teachers will result in a higher level of likability.

## 2. Materials and Methods

### 2.1. Pre-Test: The Setting of Social Situations

To illustrate the types of social situations adolescents find themselves in with the presence of a variety of others, we first examined intimacy and hierarchy as experienced by adolescents and others, including classmates, good friends, and teachers [44].

#### 2.1.1. Participants of Pre-Test

A total of 84 adolescents were randomly selected from two public schools in Shandong Province (age range = 13–19 years; *M*_age_ = 15.64 years, *SD* = 1.66 years; 61 boys). All of them were of Han nationality, the majority ethnic group in China; 12% of the adolescents were from single-child families.

#### 2.1.2. Rating of Intimacy and Hierarchy

The adolescents were asked to use an 8-point scale, ranging from 1 (not close at all) to 8 (very close), and a 7-point scale, ranging from 1 (very much lower than me) to 8 (very much higher than me), to evaluate the degree of intimacy they felt and the hierarchical differences they perceived, respectively, with regard to classmates, good friends, and teachers. The higher the score, the higher the intimacy and the greater the difference in hierarchy.

#### 2.1.3. The Result of Social Situation Setting

The results of repeated measurement ANOVA showed significant differences in the intimacy and hierarchy associated with the relationships among the three groups, *F* (2, 93) = 78.58, *p* < 0.001, η_p_^2^ = 0.49; *F* (2, 93) = 145.37, *p* < 0.001, η_p_^2^ = 0.64. Post hoc tests showed that the adolescents were closest to their good friends (*M* = 6.65, *SD* = 1.23), followed by teachers (*M* = 5.18, *SD* = 1.78), then classmates (*M* = 4.81, *SD* = 1.15). The teacher’s place in the hierarchy was highest (*M* = 5.62, *SD* = 0.98); the difference in the hierarchy of classmates (*M* = 4.17, *SD* = 0.66) and good friends (*M* = 4.14, *SD* = 0.54) was not significant.

### 2.2. Participants

The current study included two groups of participants. The first comprised 120 adolescents selected in grades 7 to 12 from two randomly selected public middle and high schools in Shandong Province for investigation; blank and missing data were removed, and 108 adolescents were effectively tested (age range = 12–18 years; *M*_age_ = 15.40 years, *SD* = 1.64 years; 40 boys). A total of 97.2% of them were of Han nationality, the majority ethnic group in China, and 38.9% of adolescents were from single-child families.

The second group, comprising 74 teachers (age range = 23–56 years; *M*_age_ = 37.16 years, *SD* = 9.19 years), was selected for the survey and included 24 male teachers; 31 were junior high school teachers, and 43 were high school teachers. Twenty were head teachers; furthermore, 23 had been teaching for 1–5 years, nine for 6–10 years, 22 for 11–20 years, and 20 for more than 20 years. In addition, the teachers selected covered all subjects taught by junior high school teachers.

### 2.3. Measures

The “others” in this study were classmates, good friends, and teachers. We used a situation-induced approach to describe the social situation the participants would encounter (eight social situations were presented to adolescents, and four to teachers). For example, “Assuming you are in a situation where a classmate (or your good friend, your student) with whom you have a normal relationship has experienced very positive academic emotions (e.g., happiness, pride) during the study process, and he or she has suppressed positive academic emotions in your presence; nothing shows how happy he or she is at the moment”. Then, the adolescents were asked to rate the likability of classmates and good friends, and the teachers were asked to rate the likability of the student.

The likability questionnaire developed by Chaiken and Eagly [65] and revised by Schall et al. [66] was adopted for this study. The questionnaire consists of five items (e.g., “I think this classmate is friendly”, rated on a 7-point scale). The higher the score, the more liked by others. Cronbach’s α of the questionnaire was between 0.92 and 0.98 under the conditions of the suppression or expression of positive or negative academic emotions in the presence of classmates, good friends, and teachers. The results of twelve CFA analyses indicated that all eight models were saturated.

### 2.4. Procedure and Data Analysis

Data on the adolescents were collected by doctoral students in psychology. After obtaining the consent of the school, parents, and adolescents themselves, the test was carried out anonymously by the class as a unit; and the questionnaires were collected on the spot after completion, which took about 10 min in total. The data from the teachers were collected through a web questionnaire, which took approximately 5 min to complete. Repeated measurement ANOVA and paired sample *t*-test were performed using SPSS 20.0 software.

## 3. Results

### Relationship Between Adolescents’ Emotional Suppression and Expression and Likability in Situations with Classmates and Good Friends

First, we used the situation and academic emotion type as repeated measures factors and conducted ANOVA on likability for suppressing emotion. The results showed a nonsignificant main effect of the situation, *F* (1, 107) = 3.31, *p* > 0.05, η_p_^2^ = 0.03, and a significant main effect of academic emotion type, *F* (1, 107) = 15.03, *p* < 0.001, η_p_^2^ = 0.12. Post hoc tests indicated that adolescents who suppressed negative academic emotions (*M* = 4.85, *SD* = 1.27) achieved a higher level of likability than those who suppressed positive academic emotions (*M* = 4.35, *SD* = 1.46). The interaction between situation and academic emotions was also not significant, *F* (1, 107) = 1.08, *p* > 0.05, η_p_^2^ = 0.01.

Second, we used situation and academic emotion type as repeated measures factors and conducted ANOVA on likability after expressing emotion. The results showed that the main effect of the situation was significant, *F* (1, 107) = 46.23, *p* < 0.001, η_p_^2^ = 0.30. Compared to doing so in the presence of good friends (*M* = 5.29, *SD* = 1.20), adolescents who expressed emotions in the presence of their classmates achieved a lower level of likability (*M* = 4.47, *SD* = 1.20). Neither the main effect of academic emotion type nor the interaction between situation and academic emotion was significant, *F* (1, 107) = 0.72, *p* > 0.05, η_p_^2^ = 0.01, *F* (1, 107) = 1.62, *p* > 0.05, η_p_^2^ = 0.02.

Finally, a paired sample *t*-test was conducted to investigate the likability of adolescents after suppressing and expressing positive and negative academic emotions in the presence of classmates and good friends. The results showed no significant difference between the likability of adolescents after suppressing and expressing positive academic emotions (*M* = 4.20, *SD* = 1.62; *M* = 4.57, *SD* = 1.45) in the presence of classmates, *t* (107) = −1.93, *p* > 0.05, Cohen’s d = 0.24; when experiencing negative academic emotions, likability after emotional suppression (*M* = 4.81, *SD* = 1.39) was significantly higher than that after emotional expression (*M* = 4.36, *SD* = 1.56), *t* (107) = 2.24, *p* < 0.05, Cohen’s d = 0.31. In the presence of good friends, when adolescents experienced positive academic emotions, likability after emotional suppression (*M* = 4.50, *SD* = 1.66) was significantly lower than that after emotional expression (*M* = 5.29, *SD* = 1.35), *t* (107) = −4.36, *p* < 0.001, Cohen’s d = 0.52; when experiencing negative academic emotions, likability after emotional suppression (*M* = 4.90, *SD* = 1.55) was significantly lower than that after emotional expression (*M* = 5.28, *SD* = 1.38), *t* (107) = −2.00, *p* < 0.05, Cohen’s d = 0.26. See Table 1.

To examine the differences in teachers’ sense of the likability of adolescents who suppressed and expressed positive and negative academic emotions, we conducted a paired sample *t*-test. The results showed that adolescents who suppressed positive academic emotions (*M* = 4.45, *SD* = 1.09) were significantly less likable than those who expressed them (*M* = 5.76, *SD* = 1.12), *t* (73) = −7.63, *p* < 0.001, Cohen’s d = 1.19; however, for teachers, no significant differences emerged in the likability of adolescents who suppressed and expressed negative academic emotions (*M* = 4.70, *SD* = 1.09; *M* = 4.75, *SD* = 1.13), *t* (73) = −0.27, *p* > 0.05, Cohen’s d = 0.05. See Table 2 for details.

## 4. Discussion

We examined the relationship between adolescents’ suppression and expression of academic emotions and their likability in various social situations, specifically in the presence of classmates, good friends, and teachers. The results confirm that the social outcomes of academic emotional suppression and expression varied according to social situation, which is consistent with the strategy–situation fit theory under the flexibility of emotional regulation.

### 4.1. Relationship Between Adolescents’ Suppression and Expression of Academic Emotions and Likability in Situations with Classmates and Good Friends

First, similar to results in the area of daily life [21,43], we found that adolescents who expressed academic emotions in the presence of classmates achieved a lower level of likability compared to those who did so in the presence of good friends. In other words, expressing one’s academic emotions in the presence of others with low intimacy can lead to poor social outcomes, perhaps because individuals tend to share their emotions with people who are more responsive and more supportive [67,68]. Compared to nonintimate others, intimate others constituted a more supportive group [69]; expressing emotions in the presence of such a group brought a sense of liberation to adolescent individuals and strengthened their relationships [21,70]. On the contrary, emotional expression can be ignored, rejected, or even exploited in situations where there is no support or a lack of support [71], and a lack of friendship can lead to an unfavorable outcome [21].

Second, inconsistent with hypothesis 1, we found no significant difference in classmates’ sense of the likability of classmates who suppressed and expressed positive academic emotions in their presence, perhaps because adolescents are more competitive with their classmates than others (e.g., good friends) [72]. Suppressing and expressing their positive academic emotions in the presence of their classmates can have a double effect. For example, suppression of positive academic emotions can be understood as both consideration of the feelings of others [73], resulting in positive social outcomes, and in isolation from relationships [16]. Similarly, the expression of positive academic emotions can be understood as both a signal of affinity to others [74], whereas showing off one’s success to others [75] and the sense of superiority transmitted provokes jealousy and dislike of the individual [76,77]. Consistent with hypothesis 1, we also found that the adolescents who suppressed negative academic emotions were more liked by their classmates than those who expressed negative academic emotions. As previous research has shown, adolescents’ expression of negative academic emotions in a strong competitive relationship may be inappropriate [78] because emotions can be contagious [79], and in the academic environment, the ultimate goal of all adolescents is to achieve good academic results and successfully advance to their preferred high school or university. If individuals pass on their negative feelings to other classmates, adversely affecting the learning of other students, it may damage their social interaction and affect the level of likability others associate with them [46,74]. Conversely, in situations of a lack of social support, hiding emotions from others may be less harmful or even beneficial (e.g., promoting good interpersonal interaction) [69].

Third, consistent with hypothesis 2, the study also showed that when adolescents experience positive or negative educational emotions, good friends like emotional suppressors significantly less than adolescents who express emotions. Thus, the expression of emotions in the presence of good friends is more socially adaptive, perhaps because adolescents have relatively weaker competitive relationships with good friends [72]. In the presence of a good friend, the social constraints affecting the use of the strategy of emotional regulation are not only relatively small [42], but the behavior of expressing emotions represents trust in the other person. This feeling of intimacy will increase the connection between oneself and good friends [80], improve the liking degree of good friends for oneself, and promote one’s social adaptability [21,70]. The suppression of positive and negative emotions in the presence of good friends may isolate an individual from good friends, weaken the social connection and intimacy between two people [81], lead to poor quality of intimate relationships [49], and thus affect the degree to which friends like the individual.

### 4.2. Relationship Between Adolescents’ Suppression and Expression of Academic Emotions and Likability in Situations with Teachers

Partially consistent with hypothesis 3, teachers, who are higher on the hierarchical ladder than students, prefer adolescents who express positive academic emotions in their presence to those who suppress positive academic emotions. In the context of emphasizing education as the foundation of the country, teachers are burdened with the substantial pressure of their schools’ excessive pursuit of enrollment numbers and parents’ exaggerated expectations of their children [82]. Teachers are, therefore, eager to hear good news about students’ academic performance. If students have made progress or have met with success in their studies and experienced positive academic emotions, sharing such emotions with teachers will improve their sense of professional achievement [83] and professional effectiveness [84] and promote the generation of teachers’ positive emotional experiences [85]; thus, these students seem more likable. On another level, teachers represent authority in the eyes of students [59]. Some may be hindered by differences in hierarchy or fear of authority, causing them to avoid talking about the positive academic emotions they experienced. Such “withdrawal” behavior may affect the smooth communication between teachers and students, disadvantage teachers’ understanding of their learning, increase the sense of alienation, and hinder the establishment of close teacher–student relationships [86]. In addition, no significant difference was found for teachers’ sense of the likability of those adolescents who suppressed and expressed negative academic emotions. Students who often experienced negative academic emotions indicated that they had encountered difficulties or problems in the learning process. Similar to suppressing positive academic emotions in the presence of teachers, not telling teachers about their own negative academic emotional experience may cause teachers to perceive that those students feel distant from them [86,87] and affect the teachers’ sense of the likability of those students. Students’ expression of negative academic emotions to teachers may also convey negative emotions to them [88]. Consequently, if teachers experience degrees of work difficulties and negative emotions during the teaching process [89], students’ expression of these emotions will inevitably have a negative effect on teachers.

### 4.3. Implications

Based on the relevant theoretical viewpoints on the flexibility of emotional regulation, this study has produced conclusions on the role of social situations in adolescent emotional suppression and expression and likability, providing an empirical basis and theoretic guidance for improving adolescent likability in social situations. This study is also of important practical significance in how to increase the likability of adolescents from the perspective of peers and teachers.

First, we must recognize the role that the various levels of intimacy adolescents share with peers plays in their own likability. This requires adolescents to pay attention to the impact of their peers on the social outcomes of their use of emotional suppression and expression but also to be aware of all the possible roles played by peers. In the presence of classmates, adolescents may suppress their negative academic emotions to improve the classmates’ sense of their likability. In the presence of good friends, we recommend that adolescents express their positive and negative academic emotions in order to increase their likability among those friends.

Second, adolescents should also use academic emotional suppression or expression carefully in the presence of teachers, who are higher in the school hierarchy. To make themselves appear more likable to teachers, we recommend that adolescents express their positive academic emotions in the presence of teachers.

Third, parents and educators should consciously cultivate the ability of adolescents to flexibly regulate their emotional expression with levels of intimacy and hierarchy in mind (e.g., good friends, teachers) to improve their social adaptability and likelihood of future success in the workplace [90], as well as maximize the benefits of emotional adjustment strategies.

### 4.4. Future Directions

Given that research on academic emotion regulation is still in the preliminary stage of exploration, we must undertake an in-depth exploration to enrich and refine relevant theoretical and empirical research.

First, the results of this study must be replicated and validated with a larger sample of adolescents and teachers in different cultures to obtain more cross-cultural research evidence. Second, future researchers could explore social outcomes of adolescents’ use of emotional suppression and expression in real academic contexts rather than through a situation-induced approach. Third, more social situations could be explored; for example, what can adolescents expect when expressing or suppressing their academic emotions in the presence of their parents? What is the difference between the results achieved in the presence of the father as opposed to the mother? Thus, providing a more comprehensive perspective on the specificity of the social outcomes of emotional suppression and expression is a desirable goal.

## 5. Conclusions

In general, the results of this study emphasize the importance of social situation in the use of strategies for academic emotion regulation and verify the adaptability of emotional regulation. It also provides an insight into how to use appropriate emotional regulation strategies for adolescents in the presence of a variety of people. For example, expressing academic emotions in the presence of good friends achieves a higher level of likability than in the presence of classmates. Furthermore, in the presence of classmates, suppressing negative academic emotions garners a higher level of likability. In the company of good friends, adolescents express whatever academic emotions they are experiencing and can get higher likability. In the presence of teachers, expressing positive academic emotions can bring even higher likability.

## Figures and Tables

**Table 1:** Likability after suppression or expression of positive or negative academic emotions in the presence of classmates and good friends.

	Positive Academic Emotions (*M* ± *SD*)		Negative Academic Emotions (*M* ± *SD*)	
	Emotional Suppression	Emotional Expression	Emotional Suppression	Emotional Expression
Classmates	4.20 ± 1.62	4.57 ± 1.45	4.81 ± 1.39	4.36 ± 1.56
Good friends	4.50 ± 1.66	5.29 ± 1.35	4.90 ± 1.55	5.28 ± 1.38

**Table 2:** Likability after suppression or expression of positive or negative academic emotions in the presence of teachers.

	Positive Academic Emotions (*M* ± *SD*)		Negative Academic Emotions (*M* ± *SD*)	
	Emotional Suppression	Emotional Expression	Emotional Suppression	Emotional Expression
Teachers	4.45 ± 1.09	5.76 ± 1.12	4.70 ± 1.09	4.75 ± 1.13

## Data Availability

The data supporting this study’s findings are available from the corresponding author upon reasonable request.

## References

[1] Lonigro A., Longobardi E., Laghi F. (2023). The interplay between expressive suppression, emotional self-efficacy and internalizing behavior in middle adolescence. Child Youth Care Forum.

[2] Ye B., Zhao S., Zeng Y., Chen C., Zhang Y. (2022). Perceived parental support and college students’ depressive symptoms during the COVID-19 pandemic: The mediating roles of emotion regulation strategies and resilience. Curr. Psychol..

[3] Tasneem S.A., Panwar D.N. (2022). Emotion regulation and psychological well-being as contributors towards mindfulness among under-graduate students. Hum. Arenas.

[4] Gross J.J. (2002). Emotion regulation: Affective, cognitive, and social consequences. Psychophysiology.

[5] Gross J.J., John O.P., Barret L.F., Salovey P. (2002). Wise emotion regulation. The Wisdom of Feelings: Psychological Processes in Emotional Intelligence.

[6] Rimé B. (2009). Emotion elicits the social sharing of emotion: Theory and empirical review. Emot. Rev..

[7] Aldao A., Sheppes G., Gross J.J. (2015). Emotion regulation flexibility. Cogn. Ther. Res..

[8] Gross J.J. (1998). Antecedent- and response-focused emotion regulation: Divergent consequences for experience, expression, and physiology. J. Pers. Soc. Psychol..

[9] Liu Y., Sang B., Liu J., Gong S., Ding X. (2019). Parental support and homework emotions in Chinese children: Mediating roles of homework self-efficacy and emotion regulation strategies. Educ. Psychol..

[10] Mauss I.B., Gross J.J., Nyklicek I., Temoshok L., Vingerhoets A. (2004). Emotion suppression and cardiovascular disease: Is hiding feelings bad for your heart?. Emotional Expression and Health: Advances in Theory, Assessment, and Clinical Applications.

[11] Lange J., Crusius J. (2015). The tango of two deadly sins: The social-functional relation of envy and pride. J. Pers. Soc. Psychol..

[12] Yang Z., Hao X. (2016). The Dictionary of Psychology.

[13] Haines S.J., Gleeson J., Kuppens P., Hollenstein T., Ciarrochi J., Labuschagne I., Grace C., Koval P. (2016). The wisdom to know the difference: Strategy-situation fit in emotion regulation in daily life is associated with well-being. Psychol. Sci..

[14] Dougherty E.N., Murphy J., Hamlett S., George R., Badillo K., Johnson N.K., Haedt-Mattet A.A. (2020). Emotion regulation flexibility and disordered eating. Eat. Behav..

[15] Paul S., Pruessner L., Strakosch A.-M., Miano A., Schulze K., Barnow S. (2023). Examining the strategy-situation fit of emotion regulation in everyday social contexts. Emotion.

[16] Cameron L.D., Overall N.C. (2018). Suppression and expression as distinct emotion-regulation processes in daily interactions: Longitudinal and meta-analyses. Emotion.

[17] Chervonsky E., Hunt C. (2017). Suppression and expression of emotion in social and interpersonal outcomes: A meta-analysis. Emotion.

[18] Tsai W., Nguyen D.J., Weiss B., Ngo V., Lau A.S. (2017). Cultural differences in the reciprocal relations between emotion suppression coping, depressive symptoms and interpersonal functioning among adolescents. J. Abnorm. Child Psych..

[19] Greenaway K.H., Kalokerinos E.K. (2017). Suppress for success? Exploring the contexts in which expressing positive emotion can have social costs. Eur. Rev. Soc. Psychol..

[20] Keltner D., Haidt J. (1999). Social functions of emotions at four levels of analysis. Cogn. Emot..

[21] Pollastri A.R., Raftery-Helmer J.N., Cardemil E.V., Addis M.E. (2018). Social context, emotional expressivity, and social adjustment in adolescent males. Psychol. Men Masculinity.

[22] Bonanno G.A., Burton C.L. (2013). Regulatory flexibility: An individual differences perspective on coping and emotion regulation. Perspect. Psychol. Sci..

[23] Liu Y., Chai X., Sang B., Zhang S. (2024). Differences in the effect of adolescents’ strategies for expressing academic emotions on academic emotions and peer acceptance in competitive and cooperative situations. Front. Psychol..

[24] Zhang S., Sang B., Pan T., Liu Y., Ma M. (2017). Emotion regulation flexibility: An integrative review. J. Psychol. Sci..

[25] Bridges L.J., Denham S.A., Ganiban J.M. (2004). Definitional issues in emotion regulation research. Child Dev..

[26] Cole P.M., Martin S.E., Dennis T.A. (2004). Emotion regulation as a scientific construct: Methodological challenges and directions for child development research. Child Dev..

[27] Keltner D., Gruenfeld D.H., Anderson C. (2003). Power, approach, and inhibition. Psychol. Rev..

[28] Kalokerinos E.K., Greenaway K.H., Casey J.P. (2017). Context shapes social judgments of positive emotion suppression and expression. Emotion.

[29] Catterson A.D., Eldesouky L., John O.P. (2017). An experience sampling approach to emotion regulation: Situational suppression use and social hierarchy. J. Res. Pers..

[30] Latané B. (1981). The psychology of social impact. Am. Psychol..

[31] Morrongiello B.A., Dawber T. (2000). Mothers’ responses to sons and daughters engaging in injury-risk Behaviors on a playground: Implications for sex differences in injury rates. J. Exp. Child Psychol..

[32] Morrongiello B.A., Sedore L. (2005). The influence of child attributes and social-situational context on school-age children’s risk taking behaviors that can lead to injury. J. Appl. Dev. Psychol..

[33] Yang Z., Xie Y. (2016). Specific negative emotions and the presence of others impact on effect of prototype elicitation during insight problem solving. J. Southwest Univ. (Nat. Sci.).

[34] Gross J.J., Richards J.M., John O.P., Snyder D.K., Simpson J.A., Hughes J.N. (2006). Emotion regulation in everyday life. Emotion Regulation in Couples and Families: Pathways to Dysfunction and Health.

[35] English T., John O.P., Gross J.J., Simpson J.A., Campbell L. (2013). Emotion regulation in close relationships. The Oxford Handbook of Close Relationships.

[36] Fischer A.H., Manstead A.S., Lewis M., Haviland-Jones J.M., Barrett L.F. (2008). Social functions of emotion. Handbook of Emotions (Vol. 3).

[37] Greenaway K.H., Kalokerinos E.K., Murphy S.C., McIlroy T. (2018). Winners are grinners: Expressing authentic positive emotion enhances status in performance contexts. J. Exp. Soc. Psychol..

[38] Van Kleef G.A. (2009). How emotions regulate social life: The emotions as social information (EASI) model. Curr. Dir. Psychol. Sci..

[39] Hareli S., Hess U. (2010). What emotional reactions can tell us about the nature of others: An appraisal perspective on person perception. Cogn. Emot..

[40] Tamir M. (2016). Why do people regulate their emotions? A taxonomy of motives in emotion regulation. Pers. Soc. Psychol. Rev..

[41] English T., John O.P. (2013). Understanding the social effects of emotion regulation: The mediating role of authenticity for individual differences in suppression. Emotion.

[42] Zhou T., Shang Z., Wang D. (2016). Emotion suppression in multiple social contexts and its effects on psychosocial functioning: An investigation with Chinese samples. Asian J. Soc. Psychol..

[43] Clark M.S., Finkel E.J. (2005). Willingness to express emotion: The impact of relationship type, communal orientation, and their interaction. Pers. Relatsh..

[44] Hwang K.K. (1987). Face and favor: The Chinese power game. Am. J. Sociol..

[45] Yang K.S. (1981). Social orientation and individual modernity among Chinese students in Taiwan. J. Soc. Psychol..

[46] Liu Y., Zhang S., Sang B., Chai X. (2023). “The same from situation to situation” or “Vary from situation to situation”: The effect of social situation on the use of emotional expression strategies. Chin. J. Clin. Psychol..

[47] Sommers S. (1984). Reported emotions and conventions of emotionality among college students. J. Pers. Soc. Psychol..

[48] Staw B.M., Sutton R.I., Pelled L.H. (1994). Employee positive emotion and favorable outcomes at the workplace. Organ. Sci..

[49] Morris A.S., Silk J.S., Steinberg L., Myers S.S., Robinson L.R. (2007). The role of the family context in the development of emotion regulation. Soc. Dev..

[50] Impett E.A., Kogan A., English T., John O., Keltner D. (2012). Suppression sours sacrifice: Emotional and relational costs of suppressing emotions in romantic relationships. Personal. Soc. Psychol. Bull..

[51] English T., Lee I.A., John O.P., Gross J.J. (2017). Emotion regulation strategy selection in daily life: The role of social context and goals. Motiv. Emot..

[52] Martini T.S. (2011). Effects of target audience on emotion regulation strategies and goals. Soc. Psychol..

[53] Spear L.P. (2009). Heightened stress responsivity and emotional reactivity during pubertal maturation: Implications for psychopathology. Dev. Psychopathol..

[54] Roach A. (2018). Supportive peer relationships and mental health in adolescence: An integrative review. Issues Ment. Health Nurs..

[55] Steinberg L., Silk J.S., Bornstein M.H. (2002). Parenting adolescents. Handbook of Parenting: Children and Parenting.

[56] Mittmann G., Zehetmayer S., Schrank B. (2021). Study protocol for a randomized controlled trial to evaluate the effectiveness of a serious game targeting interpersonal emotion regulation in early adolescents. Trials.

[57] Wang Y., Hawk S.T., Zong W. (2020). Bidirectional effects between expressive regulatory abilities and peer acceptance among Chinese adolescents. J. Exp. Child Psychol..

[58] Bonanno G.A., Papa A., Lalande K., Westphal M., Coifman K. (2004). The importance of being flexible: The ability to both enhance and suppress emotional expression predicts long-term adjustment. Psychol. Sci..

[59] Hu C., Zhang D. (2004). On authority, prestige and educational influence of teachers. Theory. Pract. Educ..

[60] Xie Q., Zheng H., Jiang G., Ren Z., Fan Y., Liu J., Zhang W. (2021). The reciprocal relationships between head teachers’ negotiation management behavior and teacher-student relationship and primary school students’ externalizing problem behaviors from grades four to six: A cross-lagged study. Acta. Psychol. Sin..

[61] Wang M.T., Eccles J.S. (2012). Adolescent behavioral, emotional, and cognitive engagement trajectories in school and their differential relations to educational success. J. Res. Adolesc..

[62] Liu Z., Duan H., Liu S., Mu R., Liu S., Yang Z. (2024). Improving knowledge gain and emotional experience in online learning with knowledge and emotional scaffolding-based conversational agent. Educ. Technol. Soc..

[63] Liu N., Li Z. (2021). The effect of emotional regulation on school well-being in junior middle school students: The mediating role of teacher-student relationship. Adv. Psychol..

[64] Denham S.A., McKinley M., Couchoud E.A., Holt R. (1990). Emotional and behavioral predictors of peer status in young preschoolers. Child Dev..

[65] Chaiken S., Eagly A.H. (1983). Communication modality as a determinant of persuasion: The role of communicator salience. J. Pers. Soc. Psychol..

[66] Schall M., Martiny S.E., Goetz T., Hall N.C. (2016). Smiling on the inside: The social benefits of suppressing positive emotions in outperformance situations. Personal. Soc. Psychol. Bull..

[67] Ruan Y., Reis H.T., Clark M.S., Hirsch J.L., Bink B.D. (2020). Can I tell you how I feel? Perceived partner responsiveness encourages emotional expression. Emotion.

[68] Von Culin K.R., Hirsch J.L., Clark M.S. (2018). Willingness to express emotion depends upon perceiving partner care. Cogn. Emot..

[69] Pauw L.S., Medland H., Paling S.J., Moeck E.K., Greenaway K.H., Kalokerinos E.K., Hinton J.D.X., Hollenstein T., Koval P. (2022). Social support predicts differential use, but not differential effectiveness, of expressive suppression and social sharing in daily life. Affect. Sci..

[70] Graham S.M., Huang J.Y., Clark M.S., Helgeson V.S. (2008). The positives of negative emotions: Willingness to express negative emotions promotes relationships. Personal. Soc. Psychol. Bull..

[71] Derks D., Fischer A.H., Bos A.E. (2008). The role of emotion in computer-mediated communication: A review. Comput. Hum. Behav..

[72] Liu Y., Sang B., Chai X., Zhang S. (2023). The use of academic emotional expression strategies in adolescents: Differences between competitive and non-competitive situations. Chin. J. Clin. Psychol..

[73] Chiang W.T. (2012). The suppression of emotional expression in interpersonal context. Bull. Educ. Psychol..

[74] Van Kleef G.A. (2010). The emerging view of emotion as social information. Soc. Personal. Psychol..

[75] Cikara M., Bruneau E., Van Bavel J.J., Saxe R. (2014). Their pain gives us pleasure: How intergroup dynamics shape empathic failures and counter-empathic responses. J. Exp. Soc. Psychol..

[76] Feather N.T., Sherman R. (2002). Envy, resentment, schadenfreude, and sympathy: Reactions to deserved and undeserved achievement and subsequent failure. Personal. Soc. Psychol. Bull..

[77] Paulus A., Rohr M., Dotsch R., Wentura D. (2016). Positive feeling, negative meaning: Visualizing the mental representations of in-group and out-group smiles. PLoS ONE.

[78] Burić I., Sorić I., Penezić Z. (2016). Emotion regulation in academic domain: Development and validation of the academic emotion regulation questionnaire (AERQ). Pers. Indiv. Differ..

[79] Hatfield E., Cacioppo J.T., Rapson R.L. (1993). Emotional contagion. Curr. Dir. Psychol. Sci..

[80] Rimé B., Gross J.J. (2007). Interpersonal emotion regulation. Handbook of Emotion Regulation.

[81] Mauss I.B., Shallcross A.J., Troy A.S., John O.P., Ferrer E., Wilhelm F.H., Gross J.J. (2011). Don’t hide your happiness! Positive emotion dissociation, social connectedness, and psychological functioning. J. Pers. Soc. Psychol..

[82] Wang F., Xu N. (2017). The influence of teachers’ emotions on high school students’ English learning. New Curric..

[83] Deng R. (2016). Teachers’ professional accomplishment: Connotative meaning, source and influencing factors. Teach. Educ. Res..

[84] Nias J. (1996). Thinking about feeling: The emotions in teaching. Camb. J. Educ..

[85] Darby A. (2008). Teachers’ emotions in the reconstruction of professional self-understanding. Teach. Educ..

[86] Gao S. (2013). A Research on the Relationship Among Meta-Mood, Teacher-Student Relationship and Learning State of High School Students.

[87] Schraub E.M., Ralf S., Karlheinz S. (2011). The effect of change on adaptive performance: Does expressive suppression moderate the indirect effect of strain?. J. Change Manag..

[88] Mottet T.P., Beebe S.A. Emotional contagion in the classroom: An examination of how teacher and student emotions are related. Proceedings of the Meeting of the National Communication Association.

[89] Zhang L., Fu H., Shen P. (2019). Research on job distress, negative emotions and occupational well-being of primary and secondary school teachers—A case study of Shandong provincial data survey. Contemp. Educ. Sci..

[90] Sapiee M.L., Abdullah N.A., Halim F.W., Kasim A.C., Ibrahim N. (2024). Exploring the Impact of Emotional Intelligence on Employee Creativity: The Mediating Role of Spiritual Intelligence. J. Chin. Hum. Resour. Manag..

